# Conservative Management of Delayed Submucosal Bleeding after Per-Oral Endoscopic Myotomy: A Case Report

**DOI:** 10.15388/Amed.2024.31.2.15

**Published:** 2024-12-04

**Authors:** Martina Marrelli, Dario Biasutto, Benedetto Neri, Serena Stigliano, Citterio Nicolò, Monica Pandolfi, Gianluca Andrisani, Francesco M Di Matteo

**Affiliations:** 1 Therapeutic GI Endoscopy Unit, Fondazione Policlinico Universitario Campus Bio-Medico, University of Rome, Rome, Italy; 2 Gastroenterology Unit, Fondazione Policlinico Universitario Campus Bio-Medico, Rome, Italy

**Keywords:** POEM, hematoma, submucosal bleeding, delayed bleeding, Raktažodžiai: POEM, hematoma, pogleivio kraujavimas, uždelstas kraujavimas

## Abstract

Per-Oral Endoscopic Myotomy (POEM) is recognized as the first-line therapy for achalasia, considering its high clinical efficacy and safety. Among the most important adverse events, bleeding or hematoma in the submucosal tunnel has incidence of approximately 1%.

We describe the case of woman affected by type II achalasia, treated with POEM, who presented delayed bleeding with submucosal hematoma after starting anticoagulant therapy with subcutaneous low molecular weight heparin (LMWH).

She presented with moderate-to-severe chest pain, stable vital signs and no evidence of arterial active bleeding. Therefore, urgent esophagogastroduodenoscopy was not performed, and the patient was treated conservatively with fasting, antibiotics, LMWH discontinuation and close medical supervision. After 10 days the condition was resolved, and the patients safely discharged.

The present case report adds support to the safety and efficacy of conservative management of submucosal hematomas occurring after POEM.

## Abstract (it)

Peroralinė endoskopinė miotomija (POEM) yra pripažinta pirmos eilės achalazijos gydymo metodu, nes yra labai veiksminga ir saugi. Tarp svarbiausių nepageidaujamų jos reiškinių yra kraujavimas arba hematoma po gleivine esančiame tunelyje, kurių dažnis yra apie 1 %.

Aprašome atvejį moters, sergančios II tipo achalazija ir gydytos POEM. Pradėjus antikoaguliacinį gydymą poodiniu mažos molekulinės masės heparinu (LMWH), pasireiškė uždelstas kraujavimas su submukozine hematoma. Moteriai buvo vidutinio stiprumo ir stiprus krūtinės skausmas, stabilios gyvybinės funkcijos ir nebuvo jokių arterinio aktyvaus kraujavimo požymių. Todėl skubi ezofagogastroduodenoskopija nebuvo atliekama, o pacientė buvo gydoma konservatyviai – badavimas, antibiotikai, LMWH vartojimo nutraukimas ir atidi gydytojo priežiūra. Po 10 dienų būklė išnyko ir pacientė išrašyta.

Šis atvejo aprašymas patvirtina konservatyvaus po POEM atsiradusių poodinių hematomų gydymo saugumą ir veiksmingumą.

## Introduction

Achalasia is a rare disorder with a yearly incidence of 1 per 100.000 people. The first Per-Oral Endoscopic Myotomy (POEM) was performed in 2008 by Inoue et al. [1] and has become gradually accepted as first-line therapy for achalasia.

Adverse events include bleeding or hematoma in the submucosal tunnel with an incidence of approximately 1%.[2] The use of anticoagulants is a known risk factor for postprocedural bleeding. However, the optimal timing and the better anticoagulant to use after POEM are still not worldwide defined. Moreover, no specific recommendations are currently available regarding the optimal management of delayed submucosal bleeding occurring after POEM.

Therefore, we describe the case of young woman affected by type II achalasia, treated with POEM, who presented delayed bleeding with submucosal hematoma in 7th postoperative day (POD) after starting low molecular weight heparin treatment to treat a superficial thrombophlebitis of the right arm, and who was successfully managed conservatively.

## Case report

A 47-year-old female has been suffering from progressive dysphagia, episodes of early postprandial vomiting and epigastric pain for approximately 3 years. She was taking antihypertensive and inhalation drugs for asthma, while she was not on antithrombotic therapy. After undergoing esophagogastroduodenoscopy (EGD) and high-resolution manometry, a diagnosis of type II achalasia according to the Chicago classification V4.0 was made.[3]

The patient was referred to our Therapeutic Endoscopy Unit of the Fondazione Policlinico Campus Bio-Medico di Roma to undergo POEM treatment. The procedure was performed under general anaesthesia with a therapeutic gastroscope (EG-760CT, Fujifilm, Fujifilm Corp., Tokyo, Japan) and the HybridKnife T-type (ERBE Elektromedizin GmbH, Tübingen, Germany). A submucosal tunnel with a length of 15 cm was created, followed by selective endoscopic myotomy of the circular muscle bundles for a total length of 8 cm (5 esophageal side and 3 gastric side). Haemostasis of small visible vessels was performed with coagulation forceps (Ensure, Coagulation forceps, Micro-Tech Nanjing Co., Ltd, Hamburg, Germany) and the entry site of the tunnel was closed with the scope clips.

After POEM, according to our internal protocol, 24 hours fasting was prescribed followed by an esophagogram showing smooth contrast progression without leak or pneumothorax. The patient started oral intake with soft food without any discomfort or pain. On 3rd POD the patient was discharged with a creamy consistency diet prescribed for 7 days.

**Figure 1:**
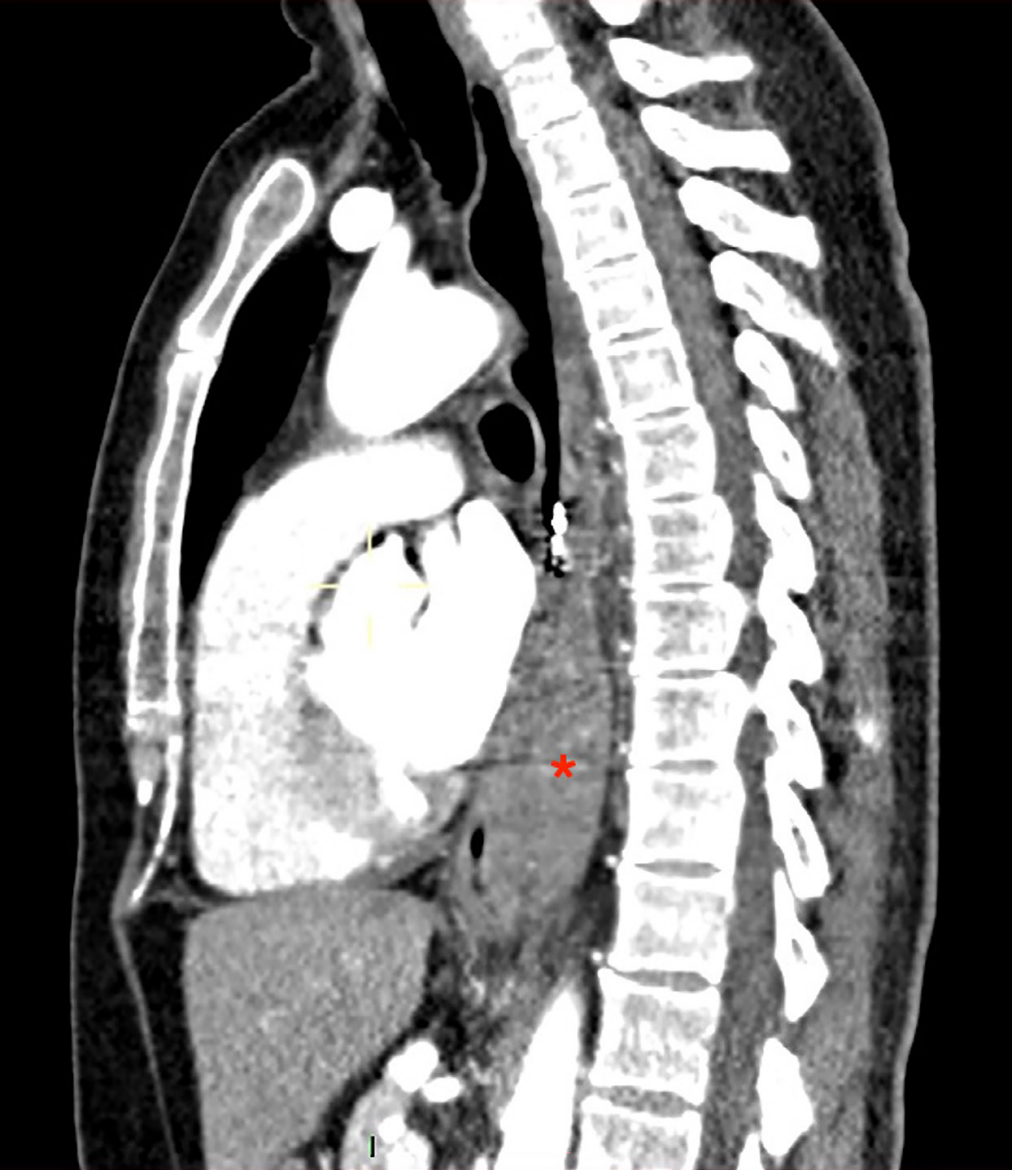
Contrast-enhanced, sagittal plane CT scan of the chest performed the 7th POD, at the time of the admission, showing the large submucosal esophageal haematoma which obliterates the esophageal lumen, without extraluminal leaks or evidence of intraluminal bleeding.

On 7th POD the patient presented to the Emergency Room of her referring hospital with moderate-to-severe epigastric and retrosternal pain began after having started Enoxaparin 6000 I.U. s.c. twice a day, as prescribed by her general practitioner to treat a superficial thrombophlebitis of the right arm. At admission, vital signs were unworrying as no tachycardia or arterial hypotension were observed. The patient underwent an urgent chest and abdomen c.e. CT scan which showed a large submucosal esophageal haematoma (8.5x3.5x6 cm), a minimal active venous bleeding into the submucosal tunnel, 2–4 cm distally the endoclips, obliteration of the esophageal lumen, without extraluminal leaks or evidence of intraluminal bleeding (Figure 1).

The patient was then referred and hospitalized at our Unit. Considering the stability of vital signs, the response to mild analgesics (paracetamol 1 g i.v.) of epigastric pain and the absence of melena or further decrease in her haemoglobin levels, we decided not to perform urgent EGD.

The anticoagulant therapy was discontinued, fasting and prophylactic antibiotic therapy with Meropenem were prescribed. After 4 days a chest c.e. CT scan was performed which showed stability of the dimensions of the submucosal hematoma, without evidence of active bleeding.

**Figure 2:**
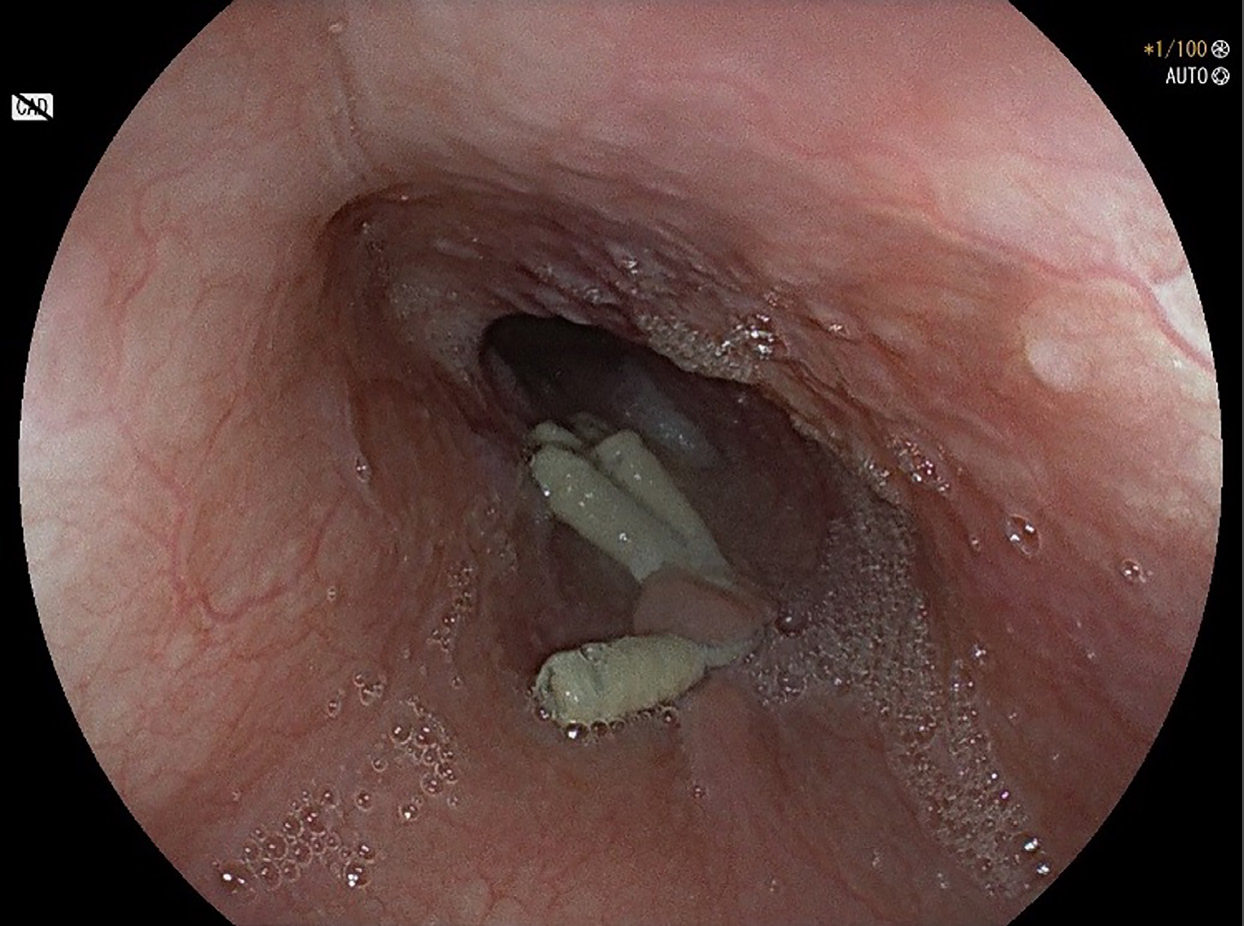
Endoscopic image of the EGD performed the 16th POD, showing metal clips in site and a bulging of the wall of the distal esophagus. The transit of the standard gastroscope through the esophago-gastric junction into the stomach was regular.

**Figure 3:**
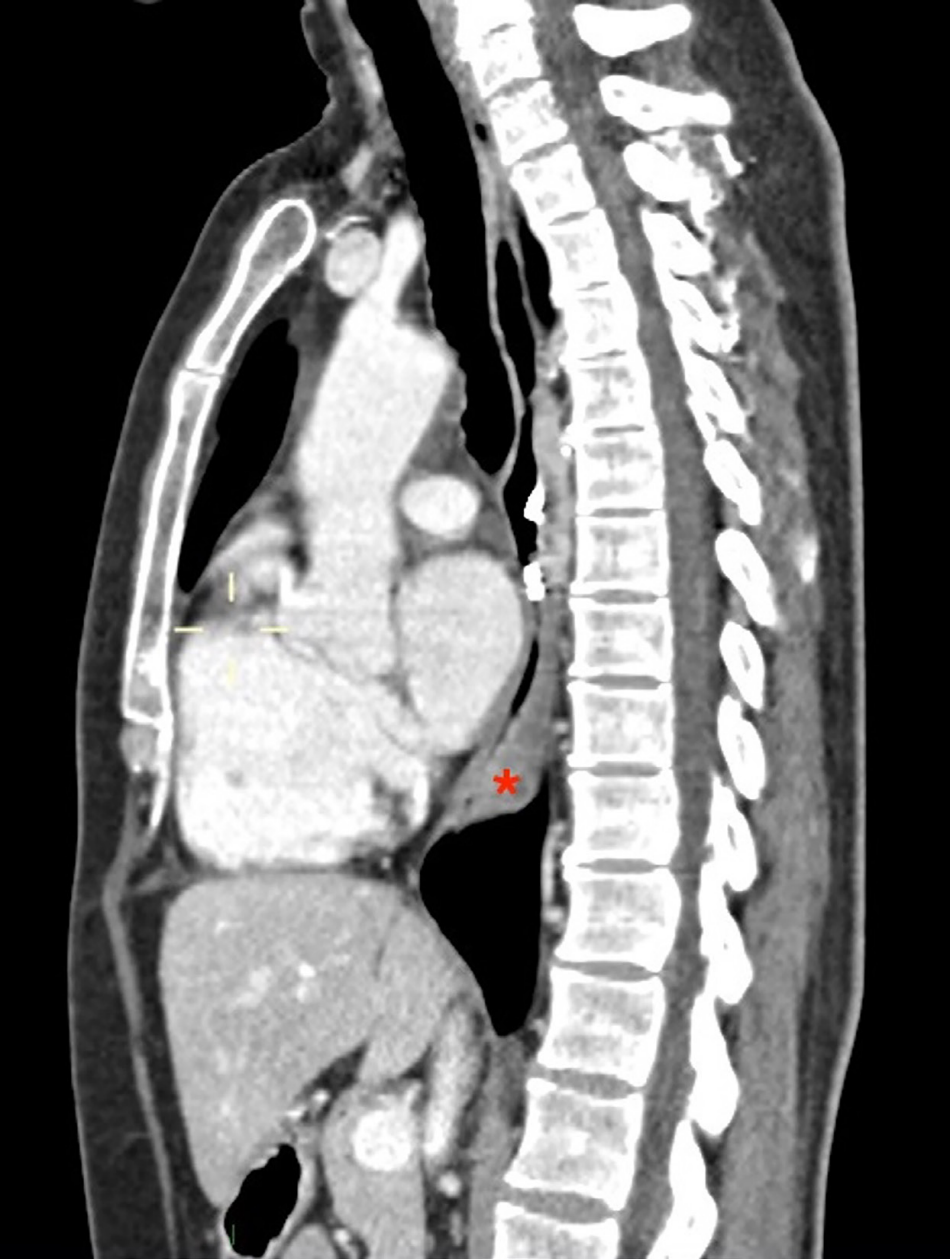
Contrast-enhanced, sagittal plane CT scan of the chest performed 1 month after POEM, showing a marked reduction of the dimensions of the submucosal esophageal haematoma. The patency of the esophageal lumen is now restored.

On 16th POD, EGD documented metal clips in site, bulging of the wall of the distal esophagus but regular transit of the standard gastroscope through the esophago-gastric junction into the stomach (Figure 2). Therefore, she started oral intake without symptoms. On 18th POD the patient was discharged. One month later, a chest CT scan was performed which showed a clear reduction in the submucosal hematoma (3x2.4x1.3 cm vs 8.5x3.5x6 cm) (Figure 3).

## Discussion

Since the first description by Inoue et al. in 2010, [1] POEM has become the first-line therapy for achalasia. The procedure consists in the creation of a submucosal tunnel from the esophagus through the cardias into the stomach. The length of the myotomy can vary according to the subtype of achalasia classified by high-resolution manometry metrics. [3] The procedure ends with closure of the entry site to the submucosal tunnel with endoclips. A 3.2% incidence of severe related adverse events including bleeding or hematoma in the submucosal tunnel, observed in 0.95% of cases (8/841), has been reported. [2]

In 2013, Li Quan-Lin et al. reported 3 out of 428 (0.7%) cases of postoperative bleeding, none on antiplatelet/anticoagulant therapy.[4] Two out of three patients presented hematemesis on the 1st and 3rd POD respectively. In the third patient, a small asymptomatic submucosal hematoma was detected at a chest CT scan, performed routinely on the 1st POD. All patients underwent endoscopic intervention. [4]

In 2021 Fujiyoshi Yusuke et al. investigated the role of second-look endoscopy after POEM, performed on the 1st POD, in a group of 497 patients.[5] They identified 7 cases of postoperative bleeding (none on antiplatelet/anticoagulant therapy), 5 with no active bleeding and small submucosal hematomas not requiring treatment and 2 with large submucosal hematomas and active bleeding into the tunnel. [5] In these patients, endoclips and blood clots in the submucosal tunnel were removed, the bleeding vessels were coagulated, and the entry site of the tunnel was closed again with endoclips. [5]

In all these bleeding cases, an endoscopic intervention was possible when the event was detected very early after the procedure. However, the progressive organization of the submucosal hematoma and the physiologic closure of the tunnel could make any endoscopic approach more difficult if performed several days after POEM.

Our internal protocol does not include routinary postoperative EGD and even if it was performed, the bleeding would probably not be found before discharge. Indeed, bleeding occurred after starting anticoagulation. Thus, even though postprocedural routinary EGD may be of help in preventing and treating post-POEM bleeding, in case of delayed bleeding it may be of little use.

Rodríguez de Santiago et al. described an increased risk of major bleeding after POEM in patients treated with antithrombotic drugs. [6] Among 126 patients on antithrombotics, bleeding occurred in 7 patients (3 on anticoagulants and 4 on aspirin) while in the 126 controls, only in 1. [6] Among the 7 cases of postprocedural bleeding, only one patient underwent heparin administration on the 1st POD. In this case, no EGD was performed, and the patient was managed only with close medical supervision. [6]

In our case, after the diagnosis of submucosal hematoma, considering the stability of vital signs, the response to mild analgesics, the absence of melena or hematemesis and the stability of hemoglobin levels, we decided not to perform urgent EGD. The patient was safely managed by fasting, prophylactic antibiotic therapy, close medical supervision and, more importantly, by discontinuing the anticoagulant therapy.

The present case report adds support to the currently available evidence suggesting a higher risk of bleeding in patients on antithrombotic treatment. However, according to our experience, when feasible, a conservative management of post-POEM hematomas appears to be safe and effective.
